# D-Mannose Regulates Hepatocyte Lipid Metabolism *via* PI3K/Akt/mTOR Signaling Pathway and Ameliorates Hepatic Steatosis in Alcoholic Liver Disease

**DOI:** 10.3389/fimmu.2022.877650

**Published:** 2022-04-07

**Authors:** Mengyao Hu, Yu Chen, Fan Deng, Bo Chang, Jialiang Luo, Lijun Dong, Xiao Lu, Yi Zhang, Zhengliang Chen, Jia Zhou

**Affiliations:** ^1^Department of Immunology, School of Basic Medical Sciences, Southern Medical University, Guangzhou, China; ^2^Department of Medical Laboratory, School of Laboratory Medicine and Biotechnology, Southern Medical University, Guangzhou, China

**Keywords:** D-mannose, hepatic steatosis, alcoholic liver disease, hepatocyte, lipid metabolism, PI3K/Akt/mTOR

## Abstract

This study investigated the protective properties and mechanisms of D-mannose against hepatic steatosis in experimental alcoholic liver disease (ALD). Drinking-water supplementation of D-mannose significantly attenuated hepatic steatosis in a standard mouse ALD model established by chronic-binge ethanol feeding, especially hepatocyte lipid deposition. This function of D-mannose on lipid accumulation in hepatocytes was also confirmed using ethanol-treated primary mouse hepatocytes (PMHs) with a D-mannose supplement. Meanwhile, D-mannose regulated lipid metabolism by rescuing ethanol-mediated reduction of fatty acid oxidation genes (PPARα, ACOX1, CPT1) and elevation of lipogenic genes (SREBP1c, ACC1, FASN). PI3K/Akt/mTOR signaling pathway was involved in this effect of D-mannose on lipid metabolism since PI3K/Akt/mTOR pathway inhibitors or agonists could abolish this effect in PMHs. Overall, our findings suggest that D-mannose exhibits its anti-steatosis effect in ALD by regulating hepatocyte lipid metabolism *via* PI3K/Akt/mTOR signaling pathway.

## Introduction

Alcoholic liver disease (ALD) is a significant health concern that causes considerable morbidity and mortality worldwide, which has become an increasingly prevalent liver disorder caused by chronic and excessive alcohol intake (1). Continued alcohol consumption can lead to a broad spectrum of hepatic lesion changes, including hepatic steatosis, inflammation, and liver injury that represent the main characteristics of ALD (2). Hepatic steatosis is the earliest form of ALD characterized by excessive fat accumulation in the liver, further developing into more severe forms of ALD, including hepatitis, fibrosis/cirrhosis, and eventually hepatocellular carcinoma and liver failure without effective treatment (3). While alcohol abstinence is the most valid therapy, targeted therapies are vital for patients who do not withdraw alcohol or with severe ALD (4). Unfortunately, there have remained no efficient therapies for ALD provided over the past few decades (5). Lipogenesis during the initial stages of ALD has been considered a significant risk factor for disease progression, suggesting that the prevention and reversal of hepatic steatosis is a potential targeted therapeutic strategy for treating ALD (6).

Although how chronic alcohol consumption causes hepatic lipid accumulation remains elusive, accumulating evidence has indicated that alcohol could affect key transcription factors that modulate lipid metabolism, such as peroxisome proliferator-activated receptor α (PPARα) and sterol regulatory element-binding protein 1c (SREBP1c), which play a crucial role in the pathogenesis of ALD (5, 7). Furthermore, alcohol exposure significantly inhibits fatty acid oxidation by inactivating PPARα, a nuclear transcription factor that regulates the mRNA expression of genes that participate in fatty-acid transportation and oxidation, such as carnitine palmitoyl transferase 1 (CPT-1), peroxisomal acyl-CoA oxidases 1 (ACOX1) (8, 9). Meanwhile, alcohol exposure can promote hepatic fatty acid synthesis by activating SREBP1c, a major transcription factor affecting *de novo* lipogenesis through up-regulation of lipogenic enzymes, including acetyl-CoA carboxylase 1 (ACC1) and fatty acid synthase (FASN) (10, 11). Additionally, emerging evidence showed that phosphatidylinositol-3-kinase (PI3K)/protein kinase B (Akt)/mammalian target of rapamycin (mTOR) signaling pathway, including PI3K/Akt pathway and its primary downstream target mTOR, plays a critical role in regulating lipid metabolism (12, 13). Furthermore, several recent studies indicated that PI3K/Akt/mTOR pathway could regulate the PPARα expression (14, 15) and SREBP1c-mediated lipogenesis (16, 17). It also reported that PI3K/Akt pathway could participate in alcohol consumption-induced fatty liver (18), and mTOR is necessary for alcohol-regulated lipid metabolism in ALD (19).

D-mannose (hereafter referred to as mannose), a 2-epimer of glucose, is present in many plants and also exists in human blood (~50 μM). It can be transported into mammalian cells but does not contribute significantly to cell bioenergetics such as glucose (20, 21). Mannose supplementation at safe supraphysiological concentrations has become an effective therapeutic strategy for patients with mannose phosphate isomerase-congenital disorder of glycosylation (MPI-CDG) (22) and recurrent urinary tract infection (UTI) (23). Emerging evidence has shown that mannose possesses potential anti-cancer (24), anti-diabetic (25), anti-fibrotic (26), anti-obesity (27), and anti-inflammation (28) bioactivities. Furthermore, mannose can be transported into mammalian cells, which further suppress proliferation/survival of tumor cells (24), promote differentiation of regulatory T cells (Tregs) (25), regulate activation of hepatic stellate cells (HSCs) (26) and macrophages (28), partially *via* tuning glucose utilization (24, 28). In addition, Jaime Chu et al. demonstrated that mannose supplementation could attenuate hepatic fibrosis induced by MPI deficiency in zebrafish and the activation of ethanol-treated human HSCs. These findings indicate the potential functions of mannose for alleviating ALD, prompting us to explore the exact role and the underlying mechanism of mannose in ALD.

In this study, we performed drinking-water supplementation of mannose in a mouse model of ALD established by chronic and binge ethanol feeding, as well as mannose treatment on primary mouse hepatocytes (PMHs) in the presence of ethanol, aimed to elucidate the potential role and underlying mechanisms of mannose in ALD *in vivo* and *in vitro*. Our findings uncover a previously unrecognized protective role of mannose against hepatic steatosis in ALD. Furthermore, mannose can exert this function by regulating ethanol-induced lipid deposition in hepatocytes *via* tuning key transcription factors that control lipid metabolism, attenuating hepatic steatosis, thus alleviating ALD progression. Therefore, these data provide a whole new insight into utilizing mannose supplementation for improving fatty liver, thus ameliorating ALD progression.

## Materials and Methods

### Chemicals and Reagents

D-mannose (purity ≥ 99%, Cat.#M2069) and ethanol (purity≥ 99.8%, 51976) were purchased from Sigma-Aldrich (St. Louis, MO, USA). Rapamycin (purity = 99.30%, S1039), LY294002 (purity = 99.84%, S1105), 740 Y-P (purity = 98.38%, S7865) and MHY1485 (purity = 99.09%, S7811) were purchased from Selleck Chemicals (Houston, Texas, USA). Liquid Standard Diet (TP4020C), Lieber-DeCarli Control Liquid Diet (TP4030C), and Lieber-DeCarli Ethanol Liquid Diet (TP4030D) were supplied by TROPHIC Animal Feed High-Tech Co. Ltd (Hai’an, Jiangsu, China). The antibodies against SREBP1c (AF-6283), PPARα (AF5301), ACC1 (AF6421), P110 of PI3K (AF-5112) were all from Affinity (Ancaster, ON, Canada). Anti-CPT1A (15184-1-AP), anti-ACOX1 (10957-1-AP), anti-FASN (10624-1-AP), anti-P85 (60225-1-Ig), anti-PPARγ (16643-1-AP), anti-PPARα (15540-1-AP) used in **Figure 6D** were obtained from Proteintech (Chicago, IL, USA). The antibodies against Akt (4691), phosphor-Akt (4060), mTOR (2983), phosphor-mTOR (5536) were from Cell Signaling Technology (Danvers, MA, USA).

### Animals Experiments

C57BL/6 mice (male, 8-10 weeks old) were purchased from the Experimental Animal Center of Southern Medical University (Guangzhou, China). All mice were housed under a 12-h light/dark cycle in a specific pathogen-free animal condition with a controlled temperature (20-25°C) and humidity (50 ± 5%). All animal experiments in this study were approved by the Southern Medical University Experimental Animal Ethics Committee (No. L2020128).

The chronic-binge mouse model was established based on the methods of previous studies with minor modifications (29). Briefly, mice were fed a standard liquid diet for 3 days, then randomly divided into different groups as follows: Pair (Lieber-DeCarli control liquid diet); EtOH (Lieber-DeCarli ethanol liquid diet; ALD group); Pair+Man (Lieber-DeCarli control liquid diet supplemented with 3% (w/v) mannose); EtOH+Man [Lieber-DeCarli ethanol liquid diet supplemented with 1%, 2%, 3% (w/v) mannose (27)]. The mice in the EtOH and EtOH+Man groups were fed the Lieber-DeCarli liquid diet containing increasing 1% to 4% (w/v) ethanol for the first 6 days and then the diet with 5% ethanol for 10 days. On day 11, mice fed ethanol before were gavaged a single dose of ethanol (5 g/kg body weight, 31.5% ethanol), while mice fed control diet were gavaged isocaloric dextrin maltose. Subsequently, the mice were sacrificed nine hours post gavage. Blood samples were obtained from the eye socket. A portion of the liver tissues was fixed in 4% neutral buffered formalin solution, and the remaining liver sections were immediately stored at -80°C.

### Isolation and Culture of Primary Mouse Hepatocytes (PMHs)

Isolated primary hepatocytes from WT C57BL/6 mice (male, 8-12 weeks old) were obtained using a classical two-step *in situ* collagenase perfusion method as described previously with slight modifications (30). Briefly, the perfused liver was immediately excised and placed in a sterile dish containing RPMI 1640 medium (Gibco, United States). Then the cell suspension was filtered through a 70-μm nylon filter (BD Biosciences) and washed thrice by centrifugation at 50 × g for 3 min at 4°C. Subsequently, the cells were resuspended in the growth medium containing William’s E medium (Thermo Fisher, Carlsbad, CA, USA) supplied with 10% fetal bovine serum (FBS, Gibco, United States), 10 ng/mL epidermal growth factor (EGF, GenScript, Nanjing, China), 2 nM L-glutamine (Macklin, Shanghai, China), 200 nM insulin (Macklin, Shanghai, China) and 100 nM dexamethasone (Macklin, Shanghai, China), and then seeded on type I collagen-coated dish. After incubation at 37°C for 4 h, PMHs were collected and washed twice, and the medium was replaced with the fresh growth medium.

Cultured PMHs were treated with 200 mM ethanol (EtOH) (31, 32) or cell growth medium only (Ctrl), in the presence of different concentrations (1 mM, 2.5 mM, 5 mM, 10 mM) of mannose (Man) (26) or not for 24 h. In some experiments, inhibitors (33) or agonists (34, 35) of PI3K or mTOR (Dimethyl sulfoxide (DMSO) as control) was added two hours before ethanol exposure or mannose treatment.

### Biochemical Analysis

Serum alanine aminotransferase (ALT), aspartate aminotransferase (AST), triglyceride (TG), total cholesterol (TC), high-density lipoprotein-cholesterol (HDL-C), low-density lipoprotein-cholesterol (LDL-C) levels, and hepatic triglyceride (TG), total cholesterol (TC) contents were all measured according to the instructions of commercial assay kits from the manufacturer (Jiancheng Biotech, Nanjing, China).

### Histopathological and Immunohistochemical Staining

The paraffin-embedded liver tissue blocks (n = 3 for each group) were cut into 5 μm slices sections and stained with hematoxylin and eosin (H&E). The frozen liver tissues (n = 3 for each group) were cut into 8 μm thick sections and then stained with Oil Red O. For immunohistochemical staining, liver tissue sections were deparaffinized and placed in a citrate buffer (pH 6.0) at 100°C for 10 min to antigen repair and then exposed to 3% H_2_O_2_ for 15 min to block endogenous peroxidase activity. Subsequently, sections were blocked with 5% normal goat serum for another 1 h at room temperature followed by incubated with primary antibodies at 4°C overnight. Immuno-reactivity was detected using the corresponding HRP-conjugated secondary antibody and visualized using a diaminobenzidine kit (Beyotime Institute of Biotechnology, Shanghai, China).

### BODIPY Staining

The cellular content of neutral lipids was detected according to the manufacturer’s instructions using lipophilic fluorescence dye BODIPY 493/503 (Invitrogen, Carlsbad, CA, USA). Briefly, cells were seeded on the 12-well culture plates containing cell-climbing slices pre-coated with collagen and incubated overnight. Cells were washed with Phosphate Buffered Saline (PBS) and fixed with 4% paraformaldehyde for 20 min at room temperature. Subsequently, cells were stained with 1 μg/mL BODIPY 493/503 dye for 30 min at 37°C, the nuclei were counterstained with 1 μg/mL Hoechst (CST) for 10 min. Then the slices were mounted on microscope glass slides and imaged immediately with a laser scanning microscope system (Nikon Eclipse Ni, Tokyo, Japan).

### Western Blotting Analysis

The protein of PMHs was homogenized in RIPA buffer containing protease inhibitor (Beyotime Institute of Biotechnology, Shanghai, China). Subsequently, the protein concentrations were measured using a BCA protein assay kit (Beyotime Institute of Biotechnology). Equivalent amounts of protein were separated by SDS-PAGE and then transferred onto polyvinylidene fluoride membranes (Millipore, Billerica, MA, USA). The membrane was blocked with 5% bovine albumin (BSA) in Tris-buffered saline containing 0.05% Tween 20 and then incubated with the specific primary antibodies, followed by HRP-conjugated secondary antibody incubation. And the target proteins were visualized with enhanced chemiluminescence (Thermo Fisher, Carlsbad, CA, USA). The intensity of the protein band was quantified using ImageJ software.

### Quantitative Real-Time PCR Analysis

The total RNA was extracted using TRIzol reagent (TransGene Biotech, Beijing, China) and then transcribed into cDNA using TranScript All-in-One First-Strand cDNA Synthesis SuperMix (TransGene Biotech), as instructed by the manufacturer. Real-time PCR was performed with an Eppendorf Realplex PCR system using TransStart Tip Green qPCR SuperMix (TransGene Biotech). The mRNA expression was normalized to the expression of the housekeeping gene β-actin. All primer sequences presented in **Table 1** were from PrimerBank (36) and synthesized by Huada Gene Technology Co., Ltd (Shenzhen, China).

**Table 1:** Primers used for real-time qRT-PCR.

Gene	Primer	Sequence (5'-3')
PPARα	Forward Reverse	AACATCGAGTGTCGAATATGTGG CCGAATAGTTCGCCGAAAGAA
CPT1	Forward Reverse	TGGCATCATCACTGGTGTGTT GTCTAGGGTCCGATTGATCTTTG
ACOX1	Forward Reverse	TAACTTCCTCACTCGAAGCCA AGTTCCATGACCCATCTCTGTC
SREBP1c	Forward Reverse	TGACCCGGCTATTCCGTGA CTGGGCTGAGCAATACAGTTC
ACC1	Forward Reverse	CTCCCGATTCATAATTGGGTCTG CTCCCGATTCATAATTGGGTCTG
FASN	Forward Reverse	GGAGGTGGTGATAGCCGGTAT TGGGTAATCCATAGAGCCCAG
β-actin	Forward Reverse	GTGACGTTGACATCCGTAAAGA GCCGGACTCATCGTACTCC

### Statistical Analysis

All data were expressed as mean ± SEM. Statistical significance was determined by the unpaired two-tailed *t*-test using GraphPad Prism 8.0 software (San Diego, CA, USA). Differences were considered statistically significant at *p* < 0.05.

## Results

### Mannose Supplement Alleviates Hepatic Steatosis in ALD

**Figure 1:**
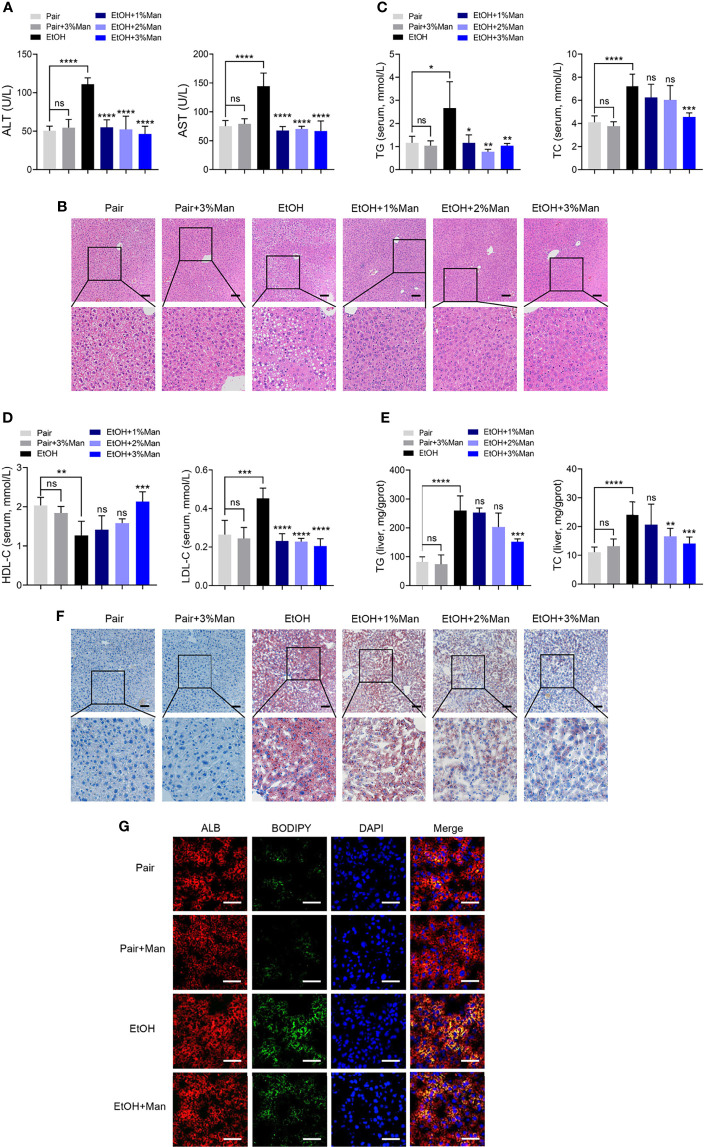
Mannose supplement alleviates hepatic steatosis in ALD. Mice were fed control diet (Pair) or ethanol diet (EtOH) supplemented with/without 1%, 2%, 3% (w/v) mannose (Man) (n = 6 for each group). **(A)** Serum ALT and AST activities were assessed. **(B)** Histologic analysis of liver sections using H&E staining. Scale bars = 100 μm. **(C, D)** Serum TG, TC, HDL-C and LDL-C levels, and **(E)** hepatic TG and TC contents were determined. **(F)** Representative images of Oil Red O staining on liver sections. Scale bars = 100 μm. **(G)** Co-localization of neutral lipids (Green) and hepatocyte markers ALB (Red) in the liver sections were evaluated by immunofluorescence staining (n = 3). Scale bars = 25μm. Data are presented as the means ± SEM and analyzed with the unpaired two-tailed *t*-test. *p < 0.05, **p < 0.01, ***p < 0.001, ****p < 0.0001, ns, not significant, compared with the EtOH group.

To address the role of mannose supplement in ALD, we investigated the degree of liver injury and hepatic steatosis in pair-fed mice (Pair) or a chronic-binge ethanol feeding mouse model of ALD (EtOH), along with drinking-water supplemented with different concentrations of mannose (Man). As shown in **Figure 1A** and **Table S1**, enzymatic assays demonstrated that mannose administration significantly reduced serum ALT and AST levels that elevated in chronic-binge ethanol-fed mice. The H&E staining of liver sections showed that ethanol-fed mice displayed extensive hepatic injuries and steatosis, which were markedly attenuated by mannose administration (**Figure 1B**). Furthermore, alcohol consumption substantially elevated TG, TC, and LDL-C levels but reduced HDL-C level in serum or liver tissue, which could be significantly inhibited by oral mannose (3%) supplement (**Figures 1C–E** and **Table S1**). Further Oil Red O staining of liver tissue sections revealed that mannose remarkably reduced ethanol-induced hepatic lipid deposits, and 3% mannose has the most significant effect (**Figure 1F**). Additionally, we performed immunofluorescence staining of liver tissue sections with BODIPY and found that mannose co-administration markedly reduced lipid deposits that present predominantly in hepatocytes upon ethanol administration (**Figure 1G**). Collectively, these results indicated a potential protective role of mannose against hepatic steatosis in ALD.

### Mannose Treatment Attenuates Ethanol-Induced Lipid Accumulation in Primary Mouse Hepatocytes

**Figure 2:**
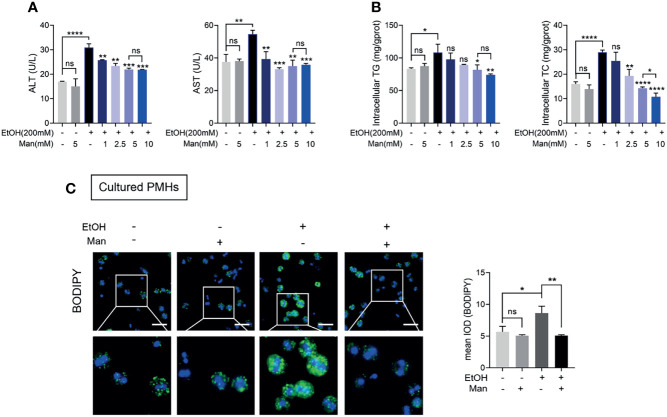
Mannose treatment attenuates ethanol-induced lipid accumulation in PMHs. PMHs isolated from WT mice were stimulated by 200 mM ethanol (EtOH) with different concentrations of mannose (Man) for 24 h (n = 3). **(A)** The ALT and AST activities in the culture supernatant were measured. **(B)** The intracellular TG and TC contents were determined. **(C)** The content of neutral lipids was detected by double staining with BODIPY 493/503 dye and Hoechst in PMHs (cultured with 5 mM mannose). Scale bars = 50 μm. Data are presented as the mean ± SEM of three independent experiments and analyzed with the unpaired two-tailed t-test. *p < 0.05, **p < 0.01, ***p < 0.001, ****p < 0.0001, ns, not significant, compared with the EtOH group.

Building upon the above findings in the mouse ALD model, we explored the exact role of mannose on hepatocytes *in vitro*. Therefore, we utilize an *in vitro* model of ALD established using ethanol-treated PMHs (32, 37, 38). Since most of the studies about the mannose supplement, mannose was added concurrently with other drugs or stimuli (24, 25), we simultaneously treated the PMHs with ethanol and indicated concentrations of mannose. Consistent with the above results *in vivo*, we demonstrated that mannose treatment significantly reduced serum ALT and AST activities and intracellular TG and TC levels in PMHs, which notably increased upon ethanol stimulation (**Figures 2A, B** and **Table S2**). Moreover, this effect was dose-dependent but with an effective plateau or saturation at concentrations higher than 5 mM (**Figures 2A, B** and **Table S2**). Further cellular staining with BODIPY 493/503 lipophilic fluorescent dye showed that ethanol notably elevated cellular neutral lipid contents deposited within lipid droplets in cultured PMHs, which significantly reduced upon mannose treatment (**Figure 2C**). These findings indicated that mannose could attenuate ethanol-induced lipid accumulation in PMHs.

### Mannose Suppresses Ethanol-Mediated Reduction of Hepatocyte Fatty Acid Oxidation

**Figure 3:**
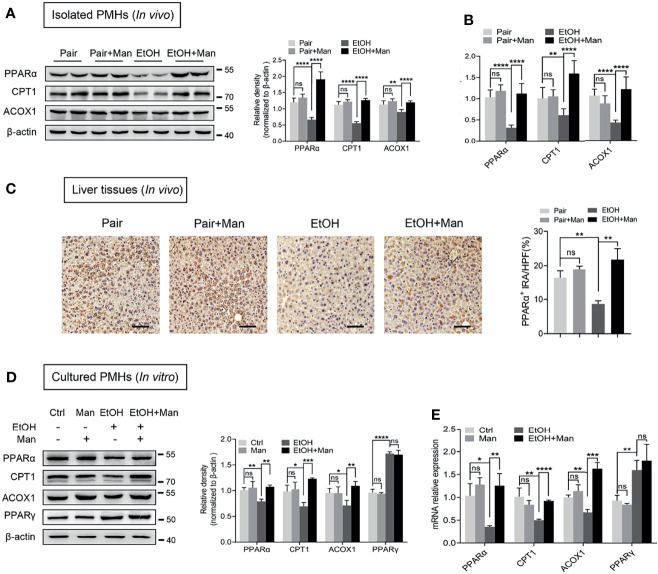
Mannose suppresses ethanol-mediated reduction of hepatocyte fatty acid oxidation. **(A–C)** PMHs were extracted from mice (n = 6 for each group) fed the control diet (Pair) or ethanol diet (EtOH) supplemented with/without 3% (w/v) mannose (Man). The protein **(A)** and mRNA **(B)** levels of PPARα, CPT1, ACOX1 were evaluated by Western blotting and qRT-PCR, respectively. **(C)** Representative images of PPARα staining on the liver sections (n = 3). Scale bars = 100 μm. **(D, E)** PMHs from WT mice were stimulated by 200 mM ethanol (EtOH) with/without 5 mM mannose (Man) for 24 h, PMHs with cell culture medium as control (Ctrl). The protein **(D)** and mRNA **(E)** levels of PPARα, CPT1, ACOX1 and PPARγwere determined (n = 3). Data are expressed as the mean ± SEM of three independent experiments. *p < 0.05, **p < 0.01, ***p < 0.001, ****p < 0.0001, ns, not significant, unpaired two-tailed *t*-test.

Considering the above results showing that mannose attenuates ethanol-induced hepatocyte lipid accumulation in ALD, we were intrigued to clarify the underlying mechanisms. Firstly, we examined the mRNA levels and protein expression of crucial fatty acid oxidation (FAO)-related genes associated with lipid metabolism. As shown in **Figures 3A, B**, we observed notably reduced protein and mRNA levels of PPARα, a key controller of FAO (39), and its downstream FAO-related genes (ACOX1, CPT1) in isolated PMHs from ethanol-fed mice than that from pair-fed controls. However, oral mannose supplement significantly increased PPARα, ACOX1 and CPT1 levels in freshly isolated PMHs from ethanol-fed mice (**Figures 3A, B**). Further immunohistochemistry analysis also showed marked elevation of PPARα expression in liver sections from ethanol-fed mice upon mannose administration, although its expression notably decreased during ethanol feeding (**Figure 3C**). Similar results showed that mannose significantly increased protein and mRNA levels of PPARα, ACOX1 and CPT1 in PMHs upon ethanol treatment *in vitro* (**Figures 3D, E**). We also evaluated the effect of mannose on PPARγ, a nuclear receptor superfamily of ligand-inducible transcription factors involved in fatty acid uptake (40, 41). While the protein and mRNA levels of PPARγ in PMHs elevated upon ethanol stimulation, they were comparable between the mannose treated or untreated PMHs *in vitro* (**Figure 3D**). These data suggest that mannose might attenuate ethanol-induced lipid accumulation in hepatocytes *via* regulating lipid metabolism by upregulating fatty acid β-oxidation.

### Mannose Inhibits Ethanol-Induced Hepatocyte Lipogenesis

**Figure 4:**
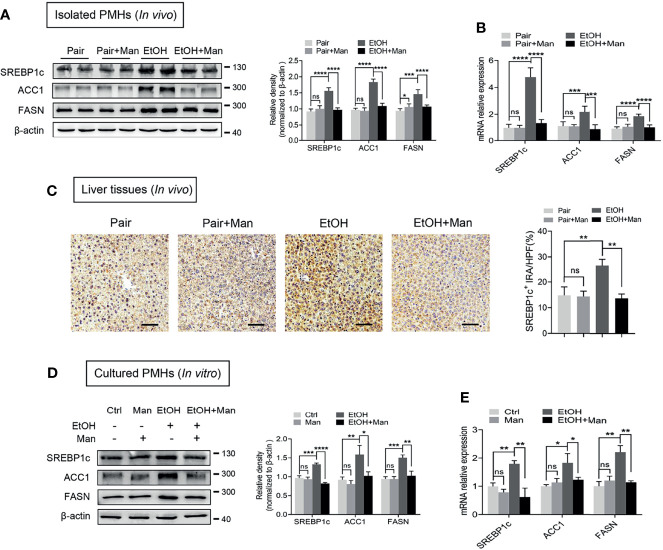
Mannose inhibits ethanol-induced hepatocyte lipogenesis. **(A-C)** Isolated PMHs were obtained from mice fed the control diet (Pair) or ethanol diet (EtOH) supplemented with or without 3% (w/v) mannose (Man). The protein **(A)** and mRNA **(B)** levels of SREBP1c, ACC1 and FASN were evaluated (n = 6). **(C)** Representative images of SREBP1c staining on the liver sections (n = 3). Scale bars = 100 μm. **(D, E)** PMHs from WT mice were stimulated by 200 mM ethanol (EtOH) with/without 5 mM mannose (Man) for 24 h, PMHs with cell culture medium as control (Ctrl). The protein **(D)** and mRNA **(E)** levels of lipogenic enzyme genes SREBP1c, ACC1, and FASN were evaluated (n = 3). Data are expressed as the means ± SEM of three independent experiments. *p < 0.05, **p < 0.01, ***p < 0.001, ****p < 0.0001, ns, not significant, unpaired two-tailed *t*-test.

Besides fatty acid oxidation, alcohol-induced hepatic lipid accumulation is also regulated by lipogenesis (42). Therefore, we next investigated whether mannose disturbs the alcohol-induced *de novo* lipogenesis in hepatocytes. As shown in **Figures 4A, B**, oral mannose supplementation could reverse the alcohol-induced elevation of protein and mRNA levels of crucial lipogenic enzyme SREBP1c and its downstream lipogenic genes (ACC1, FASN) in isolated PMHs. Further immunohistochemistry staining determined notably reduced SREBP1c expression in liver tissue sections from ethanol-fed mice upon mannose administration compared to that without mannose supplement (**Figure 4C**). Accordantly, we demonstrated that *in vitro* mannose treatment could eliminate the ethanol-induced increased protein and mRNA levels of SREBP1c, ACC1 and FASN in cultured PMHs (**Figures 4D, E**). Therefore, these data suggest that mannose might ameliorate ethanol-induced hepatocyte lipid accumulation in ALD by regulating lipid metabolism by inhibiting lipogenesis.

### Mannose Suppresses Ethanol-Induced Activation of PI3K/Akt/mTOR Signaling Pathway in Hepatocytes

**Figure 5:**
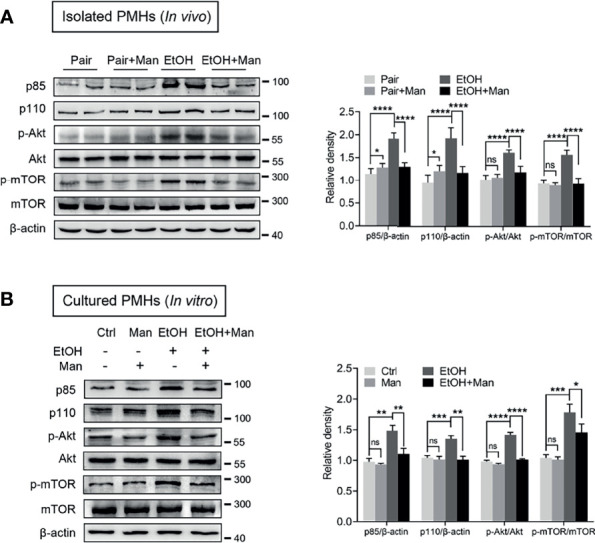
Mannose suppresses ethanol-induced activation of PI3K/Akt/mTOR signaling pathway. **(A)** Isolated PMHs were obtained from mice fed the control diet (Pair) or ethanol diet (EtOH) supplemented with or without 3% (w/v) mannose (Man). The expression levels of PI3K-p85, PI3K-p110, p-Akt, Akt, p-mTOR and mTOR in isolated PMHs were analyzed by Western blotting (n = 6). **(B)** PMHs from WT mice were stimulated by 200 mM ethanol (EtOH) with/without 5 mM mannose (Man) for 24 h, PMHs with culture medium as control (Ctrl). PI3K-p85, PI3K-p110, Akt, p-Akt, mTOR and p-mTOR levels in cultured PMHs were determined (n = 3). Data are expressed as the mean ± SEM of three independent experiments. *p < 0.05, **p < 0.01, ***p < 0.001, ****p < 0.0001, ns, not significant, unpaired two-tailed *t*-test.

Given that PI3K/Akt/mTOR signaling pathway plays a crucial role in regulating the lipid metabolic process (12, 43), we subsequently investigated whether mannose affected PI3K/Akt/mTOR signaling pathway activation. Western blotting analysis demonstrated that mannose supplement significantly downregulated alcohol-induced elevation of PI3K expression (subunit p85, p110), as well as Akt and mTOR phosphorylation in isolated PMHs from ethanol-fed mice (**Figure 5A**). Additionally, we also observed that mannose notably downregulated the ethanol-induced increased levels of PI3K (subunit p85, p110), as well as Akt and mTOR phosphorylation in PMHs upon ethanol treatment *in vitro* (**Figure 5B**). Therefore, these data indicate that mannose suppresses the ethanol-induced PI3K/Akt/mTOR signaling pathway activation in ALD.

### Mannose Improves Ethanol-Induced Lipid Accumulation in PMHs *via* The PI3K/Akt/mTOR Pathway

**Figure 6:**
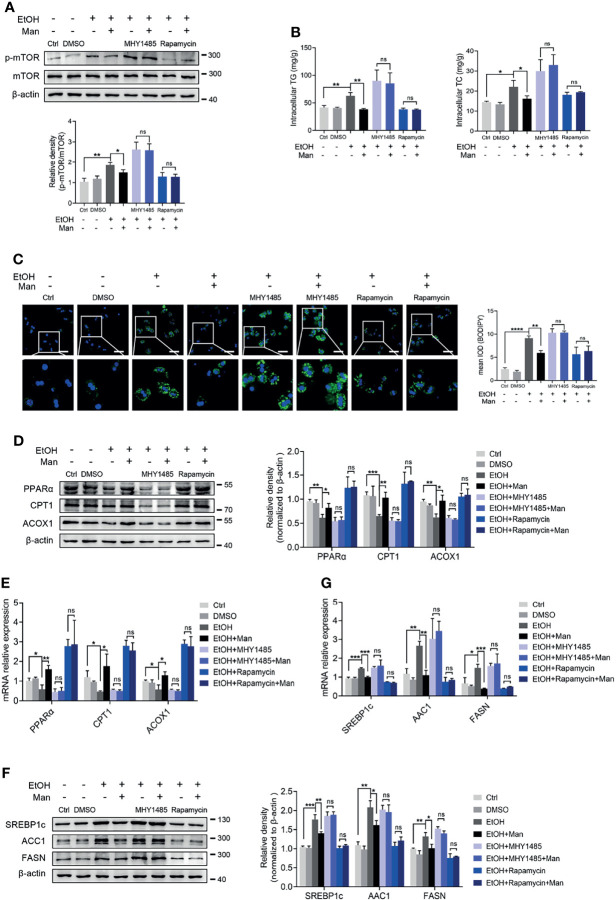
mTOR activation is involved in the mannose-mediated improvement of ethanol-induced lipid accumulation in PMHs. PMHs were pretreated with MHY1485 (mTOR agonist, 10 μM) or Rapamycin (mTOR inhibitor, 10 nM) for 2 h (DMSO as control), ahead of treatment with 200 mM ethanol (EtOH) and 5 mM mannose (Man) or culture medium (Ctrl) for 24 h (n = 3). **(A)** Western blotting was used to evaluate the p-mTOR expression in cultured PMHs. **(B)** Cellular content of TG and TC levels were determined. **(C)** The intracellular levels of neutral lipids were evaluated by BODIPY 493/503 staining assay. **(D–G)** Western blotting and qRT-PCR analysis for protein and mRNA levels of lipid-regulating genes in PMHs. Scale bars = 50 μm. Data are expressed as the mean ± SEM of three independent experiments. *p < 0.05, **p < 0.01, ***p < 0.001, ****p < 0.0001, ns, not significant, unpaired two-tailed *t*-test.

**Figure 7:**
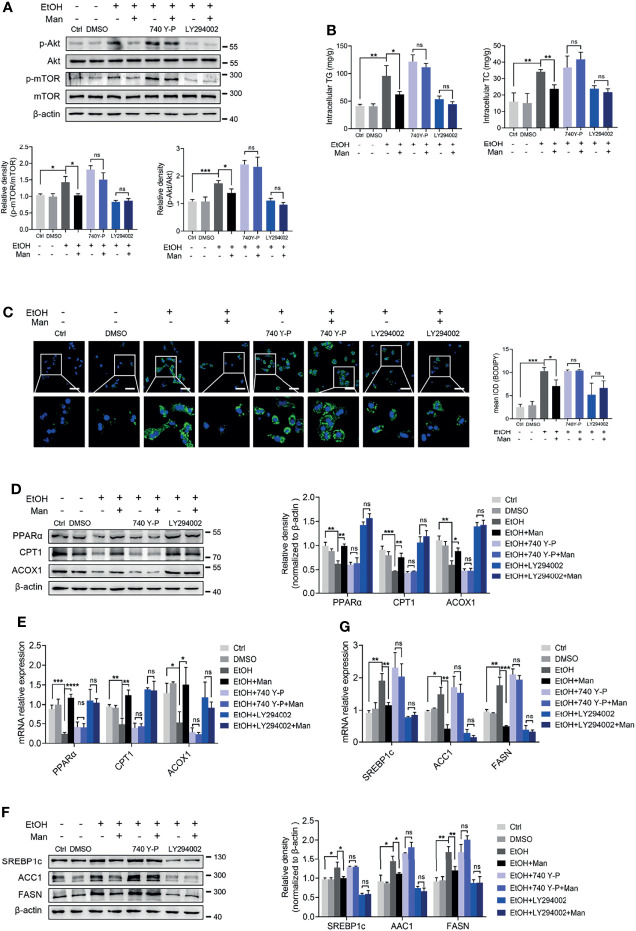
PI3K-mediated Akt/mTOR activation involves mannose-mediated improvement of ethanol-induced lipid accumulation in PMHs. PMHs from WT mice were pretreated with 740 Y-P (PI3K agonist, 20 μg/ml) or LY294002 (PI3K inhibitor, 20 μM) for 2 h (DMSO as control), ahead of treatment with 200 mM ethanol (EtOH) and 5 mM mannose (Man) or culture medium (Ctrl) for 24 h (n = 3). **(A)** Akt, p-Akt, mTOR and p-mTOR expressions were evaluated. **(B)** Cellular content of TG and TC levels were determined. **(C)** The intracellular levels of neutral lipids were determined by BODIPY 493/503 staining assay. **(D–G)** Western blotting and qRT-PCR analysis for protein and mRNA levels of lipid-regulating genes. Scale bars = 50 μm. Data are expressed as the mean ± SEM of three independent experiments. *p < 0.05, **p < 0.01, ***p < 0.001, ****p < 0.0001, ns, not significant, unpaired two-tailed *t*-test.

Our data above point to the potential involvement of the PI3K/Akt/mTOR signaling pathway in mannose-mediated alleviation of lipid accumulation driven by ethanol-mediated imbalanced lipid metabolism in hepatocytes. To test this, we pretreated PMHs with specific inhibitors or agonists of the PI3K/Akt/mTOR signaling pathway ahead of mannose with/without ethanol treatment. Western blotting analysis showed that pretreatment with the mTOR-specific inhibitor, rapamycin, did suppress mTOR phosphorylation, whereas its agonist MHY1485 could trigger mTOR phosphorylation (**Figure 6A**). However, these pretreatments could eliminate the differences of ethanol-induced hepatocyte lipid accumulation between mannose treated or untreated PMHs, as determined by comparable intracellular TG and TC levels, cellular neutral lipid contents, protein and mRNA levels of FAO-related genes (PPARα, CPT1, ACOX1) and lipogenic genes (SREBP-1, ACC1, FASN) in these cells (**Figures 6B–G**). Similar results were observed when using the PI3K inhibitor LY294002 or its agonist 740 Y-P instead of the inhibitor and agonist of mTOR (**Figure 7**). Overall, these results confirmed that PI3K/Akt/mTOR singling is responsible for the inhibitory effect of mannose on lipid accumulation in hepatocytes.

## Discussion

- Hepatic steatosis is recognized as an early symptom and a critical event during the progression of ALD, which can progress to severer liver diseases without efficient therapy (44). Thus developing effective therapeutic interventions for treating hepatic steatosis is critical to prevent further deterioration of ALD, whereas available effective target drugs are lacking (45). D-mannose, a monosaccharide widely distributed in nature, can be extracted from many plants and fruits, becoming a supplement for effective therapeutic strategies in various diseases (46). Recently, mannose supplements have been reported to treat liver-related diseases (26, 27, 47). Therefore, we were intrigued to explore the potential application of mannose supplements to prevent ALD deterioration. In the present study, while utilizing a widely used chronic-binge ethanol feeding mouse model of ALD, we discovered that oral mannose supplementation did alleviate ALD. This effect was evidenced by considerable improvement in liver injury and particular hepatic steatosis, which represented the main characteristics of ALD (1) in ethanol-fed mice upon mannose administration. A previous report indicated that mannose supplementation attenuated the liver steatosis induced by a high-fat diet when initiated early in life, suggesting a potential protective role of mannose against hepatic steatosis, which partially supports our current results (27). Consistently, we observed a similar effect of mannose in an *in vitro* model of ALD established using ethanol to stimulate primary mouse hepatocytes (32, 37, 38). Overall, our findings did emphasize the hepatoprotective role of mannose in ALD.

As we know, mannose is a 2-epimer of glucose present throughout nature, even in mammal cells (20, 21). Although most mannose is derived from glucose and further catabolized to glycosylation precursors in the cells, it can also be uptook or released by various cell types (48). It has been reported that circulating mannose levels could be influenced by metabolic disorders (21). Accumulating evidence indicated that plasma mannose levels increased in subjects with insulin resistance (IR), including diabetics (49, 50). Furthermore, several studies demonstrated that blood mannose levels are closely linked to glucose metabolism or IR and insulin secretion (50–52). However, drinking-water supplementation of supraphysiological levels of D-mannose suppressed immunopathology in mouse models of diabetes, partially by its Treg-promoting effect mediated by upregulation of integrin α_v_β_8_ and reactive oxygen species generated by increased fatty acid oxidation (25). IR and lipid metabolism dysfunction are common disorders in ALD (53, 54). Furthermore, alcohol consumption could impair the insulin signaling pathway in the liver, leading to glucose and lipid metabolism disorders, becoming vital drivers of hepatic steatosis in ALD (55, 56). Alcohol consumption also leads to defective glycosylation of lipid-carrying apolipoproteins, resulting in impaired intracellular lipid and lipoprotein transport, which in turn may contribute to alcoholic hepatic steatosis (57). These data suggest potential interactions of mannose and hepatic steatosis in ALD. However, the mechanisms need to be further explored.

Excessive lipid accumulation in hepatocytes and further hepatic steatosis are critical risk factors for ALD deterioration (58). Although previous studies indicated the potential functions of mannose for alleviating ALD (26), the exact role and the underlying mechanism of mannose in this context are still unknown. In this study, we did investigate that mannose did notably attenuate the ethanol-induced elevation of lipid deposits in hepatocytes both *in vivo* and *in vitro*, indicated by reduced ethanol-induced elevation of TG and TC levels and cellular neutral lipid contents. Intriguingly, *in vitro* experiments suggest that this effect of mannose is dose-dependent, reaching a plateau at concentrations above 5 mM. Of note, this effective working concentration is much lower than that in treating cancer cells as a supplement (24). This result was partially supported by a previous study showing that mannose attenuates hepatic stellate cell activation in a dose-dependent manner with an effective plateau (26). Furthermore, we found that mannose supplement hardly changed the transcription of the principal enzymes responsible for ethanol metabolism, alcohol dehydrogenase 1 (ADH1) and aldehyde dehydrogenase 2 (ALDH2) (59), indicating that mannose might not affect ethanol metabolism (**Figure S1**). Thus, these present results suggest that mannose alleviates lipid accumulation in hepatocytes not by affecting ethanol metabolism, thus improving hepatic steatosis in ALD.

Hepatic steatosis is characterized by excessive lipid accumulation in hepatocytes due to imbalanced lipid metabolism, driven mainly by reduced FAO, whereas increased *de novo* lipogenesis (60). PPARs, as ligand-activated transcription factors belonging to the nuclear receptor (NR) superfamily, play pivotal roles in liver diseases (61). PPARα, a subtype of PPARs, is widely expressed in the liver and regulates the mRNA expression of FAO-related genes (62). CPT1 and ACOX1, representative FAO-related genes, are the rate-limiting enzymes in the mitochondrial fatty acid oxidation and peroxisomal fatty acid β-oxidation, respectively (63). Emerging data have also demonstrated that alcohol consumption reduced fatty acid oxidation in hepatocytes through inhibiting PPARα (64). Therefore, these data prompt us to investigate whether mannose affects FAO in hepatocytes in ALD models. Our present findings indicated that ethanol did inhibit FAO in PMHs, evidenced by significantly reduced transcription and expression of PPARα and its target CPT1 and ACOX1 upon alcohol treatment. However, this ethanol-induced reduction of FAO was significantly inhibited after mannose treatment. Moreover, mannose could not significantly affect the expression of PPARγ involved in fatty acid uptake (40, 41, 65), although the PPARγ levels did elevate upon alcohol exposure as reported before (66–68). Therefore, these data imply that mannose might regulate lipid metabolism *via* upregulating fatty acid β-oxidation in ALD.

Except for fatty acid β-oxidation in cells, our current study also took *de novo* lipogenesis into account. SREBP1c is a crucial transcription factor modulating *de novo* lipogenesis *via* regulating the transcriptions of lipogenic genes such as ACC1, FASN (69). It has been reported that both acute and chronic ethanol exposure results in increased expression of SREBP1c and its target lipid synthesis enzymes (70). Furthermore, SREBP1c null mice are protected from ethanol-induced hepatic steatosis (71). Indeed, our present results confirmed that alcohol treatment significantly increased SREBP1c, ACC1 and FASN levels. As expected, we found that mannose could suppress the increased transcription and expression of lipogenic genes induced by ethanol. Therefore, our present *in vivo* and *in vitro* study suggests that mannose can regulate lipid metabolism *via* inhibiting alcohol-induced *de novo* lipogenesis in ALD. Overall, our current finding provided preliminary evidence that mannose prevented the imbalanced lipid metabolism induced by ethanol intake.

Although the exact mechanism of imbalanced lipid metabolism upon chronic alcohol consumption remains elusive, several signaling pathways might be involved where their crosstalk is complicated (45). Emerging evidence shows that the PI3K/Akt/mTOR pathway plays a critical role in lipid metabolism *via* regulating FAO and lipogenesis (12, 33, 72). Furthermore, a previous study about hepatocellular carcinoma suggests that activation of the Akt/mTOR pathway could elevate the expression of FAO regulator SREBP1c and then reprogram hepatic lipid metabolism (73). Additionally, Hanqing et al. observed that the mechanistic target of rapamycin complex 1 (mTORC1, mTOR complex 1) is necessary for ethanol-induced imbalanced metabolism, driven by induction of hepatic *de novo* lipogenesis whereas suppressing fatty acid oxidation in alcohol liver disease (19). Given our current finding showing that mannose treatment markedly upregulated the ethanol-induced PI3K/Akt/mTOR signaling pathway activation, we proposed that this pathway is involved in the mannose-mediated improvement of imbalanced lipid metabolism in hepatocytes. To confirm this hypothesis, we subsequently pretreated PMHs with both inhibitors and agonists of PI3K or mTOR, ahead of ethanol exposure with/without mannose. The present results demonstrated that these inhibitors or agonists did inhibit or activate the PI3K/Akt/mTOR pathway and regulate the expression of downstream lipid metabolism-related genes, which was in line with a previous report (33). Our current results indicated that mannose treatment suppressed lipogenesis whereas enhanced fatty acid oxidation, which further attenuated lipid accumulation in PMHs exposed to ethanol. Intriguingly, these effects of mannose were abolished upon pretreatment with these inhibitors or agonists, confirming that PI3K/Akt/mTOR signaling pathway is responsible for the inhibitory effect of mannose on lipid accumulation in hepatocytes. However, it should be noted that other signaling pathways involved in lipid metabolism could not be excluded.

In summary, this study elucidates a previously unknown hepatoprotective role of mannose against hepatic steatosis in ALD progression. Additionally, mannose can exert this protective effect by reversing imbalanced lipid metabolism to alleviate hepatocyte lipid accumulation. Furthermore, mannose can modulate lipid metabolism by upregulating fatty acid oxidation whereas damping *de novo* lipogenesis *via* inhibiting PI3K/Akt/mTOR signaling pathway activation. Thus, our present data indicate that mannose might be a potential candidate to treat alcoholic liver steatosis, providing novel insights for its application in hepatic steatosis-related liver diseases.

## Data Availability Statement

The original contributions presented in the study are included in the article/**Supplementary Material**. Further inquiries can be directed to the corresponding authors.

## Ethics Statement

The animal study was reviewed and approved by the Southern Medical University Experimental Animal Ethics Committee (No. L2020128).

## Author Contributions

MH and YC are the primary investigators in this study. FD, JL, and LD participated in part of *in vivo* experiments. FD and BC participated in part of *in vitro* experiments. XL and YZ participated in part of the statistical analysis. JZ and ZC designed the study. JZ and MH wrote the manuscript. All authors contributed to the article and approved the submitted version.

## Funding

This work was supported by the National Natural Science Foundation of China (NO.81971550 and NO.82171745).

## Conflict of Interest

The authors declare that the research was conducted in the absence of any commercial or financial relationships that could be construed as a potential conflict of interest.

## Publisher’s Note

All claims expressed in this article are solely those of the authors and do not necessarily represent those of their affiliated organizations, or those of the publisher, the editors and the reviewers. Any product that may be evaluated in this article, or claim that may be made by its manufacturer, is not guaranteed or endorsed by the publisher.
