# Supplementary material for: D-Mannose Regulates Hepatocyte Lipid Metabolism via PI3K/Akt/mTOR Signaling Pathway and Ameliorates Hepatic Steatosis in Alcoholic Liver Disease
Source: Front Immunol. 2022 Apr 7;13:877650. doi: 10.3389/fimmu.2022.877650 (PMC9021718; doi:10.3389/fimmu.2022.877650)
Supplement: Supplementary file 1 [file DataSheet_1.pdf]

Supplementary materials

Primers used for real-time qRT-PCR

Gene	Primer	Sequence (5'-3')
ADH1	Forward	CCATCGAGGACATAGAAGTCGC
	Reverse	TGGTTTCACACAAGTCACCCC
ALDH2	Forward	TTCCCACCGTCAACCCTTC
	Reverse	CCAATCGGTACAACAGCCG

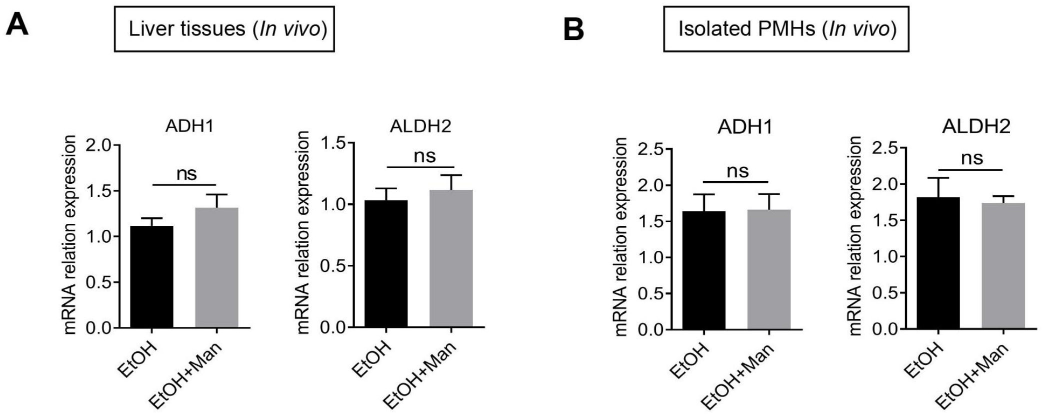

Figure S1. Mannose has no effect on key enzymes of ethanol metabolism. (A) Liver tissues were obtained from mice fed the ethanol diet (EtOH) supplemented with or without 3% (w/v) mannose (Man). The mRNA levels of ADH1, ALDH2 were evaluated by qRT-PCR (n = 6). (B) Isolated PMHs were obtained from mice fed the ethanol diet (EtOH) supplemented with or without 3% (w/v) mannose (Man). The mRNA levels of ADH1, ALDH2 were evaluated qRT-PCR (n = 5). Data are expressed as the mean ± SEM of three independent experiments. ns, not significant, unpaired two-tailed t-test.

Table S1. Effects of mannose on Biochemical Parameters in ALD

Group	Pair	Pair+3%Man	EtOH	EtOH+1%Man	EtOH+2%Man	EtOH+3%Man
ALT (U/L)	50.51±2.42	54.58±4.31	111.00±3.40#	55.01±3.40*	52.30±7.02*	46.32±4.10*
AST (U/L)	75.15±4.00	79.25±3.62	144.40±9.27#	67.66±2.885*	70.45±1.86*	66.94±6.98*
TG (serum, mmol/L)	1.17±0.11	1.03±0.09	2.66±0.47#	1.16±0.14*	0.77±0.04*	1.03±0.04*
TC (serum, mmol/L)	4.11±0.23	3.76±0.16	7.21±0.43#	6.23±0.47	6.01±0.51	4.55±0.14*
HDL-C (serum, mmol/L)	2.03±0.08	1.84±0.07	1.27±0.15#	1.42±0.15	1.58±0.04	2.13±0.10*
LDL-C (serum, mmol/L)	0.26±0.03	0.24±0.02	0.45±0.02#	0.23±0.02*	0.23±0.01*	0.21±0.02*
TG (liver, mg/gprot)	81.99±7.12	74.46±13.04	259.90±6.32#	253.20±6.32	203.20±19.65	152.20±3.95*
TC (liver, mg/gprot)	11.10±0.60	13.18±0.86	24.09±1.56#	20.73±2.47	16.70±0.94*	14.19±0.77*

Data are expressed as the means ± SEM and analyzed with unpaired two-tailed t-test ($^{\#}p < 0.05$: significantly different from the Pair group, $^{*}p < 0.05$: significantly different from the EtOH group).

Table S2. Effects of mannose on Biochemical Parameters in Cultured PMHs

Group	ALT (U/L)	AST (U/L)	Intracellular TG (mg/gprot)	Intracellular TC (mg/gprot)
Ctrl	16.99±0.08	37.63±2.63	83.06±0.95	16.00±0.52
Man (5 mM)	15.04±1.78	37.99±0.77	87.28±2.37	13.93±0.97
EtOH	30.89±0.89#	54.56±1.40#	108.20±7.37#	28.97±0.51#
EtOH+Man (1 mM)	25.73±0.09*	39.28±2.64*	97.54±5.67	25.40±2.07
EtOH+Man (2.5 mM)	23.33±0.61*	33.09±0.50*	89.29±0.43	19.28±0.29*
EtOH+Man (5 mM)	21.95±0.29*	34.99±2.12*	81.52±4.41*	14.28±0.29*
EtOH+Man (10 mM)	21.78±0.08*	35.79±0.50*	73.95±0.76*	10.78±0.87*

Data are expressed as the means ± SEM and analyzed with unpaired two-tailed t-test ($^{\#}p < 0.05$: significantly different from the Ctrl group, $^{*}p < 0.05$: significantly different from the EtOH group).
